# Further evidence of a cumulative effect of social disadvantage on risk of psychosis

**DOI:** 10.1017/S0033291716002993

**Published:** 2016-12-05

**Authors:** S. A. Stilo, C. Gayer-Anderson, S. Beards, K. Hubbard, A. Onyejiaka, A. Keraite, S. Borges, V. Mondelli, P. Dazzan, C. Pariante, M. Di Forti, R. M. Murray, C. Morgan

**Affiliations:** 1 Department of Psychosis Studies, Institute of Psychiatry, Psychology & Neuroscience, King's College London, London, UK; 2 National Institute for Health Research (NIHR) Mental Health Biomedical Research Centre at South London and Maudsley NHS Foundation Trust and King's College London, London, UK; 3 Department of Health Service & Population Research, Institute of Psychiatry, Psychology & Neuroscience, King's College London, London, UK; 4 Department of Psychological Medicine, Institute of Psychiatry, Psychology & Neuroscience, King's College London, London, UK; 5 MRC Social, Genetic & Developmental Psychiatry Centre, Institute of Psychiatry, Psychology & Neuroscience, King's College London, London, UK

**Keywords:** Causality, environment, epidemiology, reverse causation, schizophrenia, social adversity, unemployment

## Abstract

**Background:**

A growing body of evidence suggests that indicators of social disadvantage are associated with an increased risk of psychosis. However, only a few studies have specifically looked at cumulative effects and long-term associations. The aims of this study are: To compare the prevalence of specific indicators of social disadvantage at, and prior to, first contact with psychiatric services in patients suffering their first episode of psychosis and in a control sample. To explore long-term associations, cumulative effects, and direction of effects.

**Method:**

We collected information on social disadvantage from 332 patients and from 301 controls recruited from the local population in South London. Three indicators of social disadvantage in childhood and six indicators of social disadvantage in adulthood were analysed.

**Results:**

Across all the domains considered, cases were more likely to report social disadvantage than were controls. Compared with controls, cases were approximately two times more likely to have had a parent die and approximately three times more likely to have experienced a long-term separation from one parent before the age of 17 years. Cases were also more likely than controls to report two or more indicators of adult social disadvantage, not only at first contact with psychiatric services [odds ratio (OR) 9.5], but also at onset of psychosis (OR 8.5), 1 year pre-onset (OR 4.5), and 5 years pre-onset (OR 2.9).

**Conclusions:**

Greater numbers of indicators of current and long-term exposure are associated with progressively greater odds of psychosis. There is some evidence that social disadvantage tends to cluster and accumulate.

## Introduction

Evidence for the effects of social factors on psychosis is accumulating (Corcoran *et al.* 2003; Morgan *et al.* 2010; Howes & Murray, 2014). Reviews of the literature suggest that being from a minority ethnic group (Boydell *et al.* 2001; Kirkbride *et al.* 2012) and having been subjected to childhood trauma (Read *et al.* 2005; Morgan & Fisher 2007*a*; Bendall *et al.* 2008) are associated with an increased risk of psychosis. In addition, there is emerging evidence that specific indicators of social disadvantage are related to psychosis. Experiences of separation or loss of a parent in childhood (Agid *et al.* 1999; Morgan *et al.* 2007*b*; Rubino *et al.* 2009; Stilo *et al.* 2013) as well as later social disadvantage in the form of living alone (Bechdolf *et al.* 2005; Drukker *et al.* 2006; Morgan *et al.* 2008; Turner *et al.* 2009; Ramsay *et al.* 2012), being single, divorced, separated (Tien & Eaton 1992; Agerbo *et al.* 2004; Drukker *et al.* 2006; Monte *et al.* 2008; Morgan *et al.* 2008; Pelayo-Terán *et al.* 2008; Turner *et al.* 2009; Ramsay *et al.* 2012; ) and being unemployed (Agerbo *et al.* 2004; Bechdolf *et al.* 2005; Drukker *et al.* 2006; Monte *et al.* 2008; Morgan *et al.* 2008; Pelayo-Terán *et al.* 2008; Reininghaus *et al.* 2008; Turner *et al.* 2009; Dewa *et al.* 2012; Ramsay *et al.* 2012; Tandberg *et al.* 2012) are more common among those with a first-episode psychosis.

Although many studies have looked at the association between single indicators of social disadvantage and psychosis, only a few studies have specifically looked at cumulative effects and long-term associations (Agerbo *et al.* 2004; Morgan *et al.* 2008; Stilo *et al.* 2013).

Agerbo and colleagues, using data from three Danish population-based registers, identified a total of 5341 cases diagnosed with schizophrenia at their first admission in the period 1970–1999 and matched each individual with 10 persons of the same sex, who were born in the same year, and who were never admitted. They found that up to 15 years before admission, individuals who were not fully employed or self-employed and those who were single, were more likely to be later admitted with schizophrenia (Agerbo *et al.* 2004). Morgan and colleagues, in the Aetiology and Ethnicity in Schizophrenia and Psychoses (AESOP) study, a multi-centre case-control study examining data on 390 cases and 391 controls, found that, compared with controls, cases were three times more likely to live alone, three times more likely to be single, four times more likely to be unemployed, and two times more likely to live in rented accommodation (Morgan *et al.* 2008). These associations were of similar magnitude and still statistically significant 1 year prior to contact with psychiatric services (*p* < 0.05) and after adjusting for age, gender, ethnicity and study centre (Morgan *et al.* 2008). Furthermore, in our previous study, the Genetic and Psychosis (GAP) study, social disadvantage was associated with greater risk of psychosis not only at first contact with psychiatric services[odds ratio (OR) 9.03, 95% confidence interval (CI) 5.60–14.58] but also 1 year prior to admission (OR 5.67, 95% CI 3.57–9.02) and 5 years prior to admission (OR 2.68, 95% CI 1.62–4.45) (Stilo *et al.* 2013). None of the studies published to date have distinguished between social circumstances at onset, before onset and at first contact with psychiatric services.

Therefore, the aims of this article are: (1) To compare the prevalence of specific indicators of social disadvantage in childhood, 5 years pre-onset of psychosis, 1 year pre-onset, at onset, and at first presentation to psychiatric services, in patients suffering their first episode of psychosis, and in a control sample. (2) To explore long-term associations, cumulative effects (dose-response associations), and direction of effects.

## Method

This research forms part of Childhood Adversity and Psychosis Study (CAPsy study) and the European Network of National Schizophrenia Networks studying Gene-Environment Interaction (EU-GEI study). This part is a case-control study of first episode psychosis, conducted over a 5-year period (1 May 2010 to 1 May 2015), aiming to identify the genetic, clinical and environmental factors involved in the development, severity, and outcome of psychotic disorders.

### Cases

Cases were individuals with a first episode of psychosis [ICD-10: F20-29; F30-33 (psychosis codings)], aged 18–64 years, resident within a clearly defined catchment area in the south-east London boroughs of Lambeth and Southwark, who presented for the first time to specialist mental health services (population aged 18–64, approximately 3 32 000). The recruitment strategy was based on contacting out-patient and in-patient services regularly, interviewing staff and reviewing clinical notes, and approaching all subjects who met the inclusion criteria. To identify cases, a team of researchers regularly checked all points of potential contact with specialist mental health services in the catchment areas. All potential cases were screened for inclusion using the Screening Schedule for Psychosis (Jablensky, 1997). Each patient meeting inclusion criteria was approached and informed consent sought. 332 patients consented to participate in the study. Exclusion criteria were age <18 or >64 years, treatment with anti-psychotic medication for an episode of psychosis outside of the study period, evidence of psychotic symptoms precipitated by an organic cause, and transient psychotic symptoms resulting from acute intoxication as defined by ICD-10 (WHO, 1992).

### Controls

During the same period, 301 controls were recruited, with the same inclusion and exclusion criteria as for cases, with the exception that controls had no history of a psychotic disorder. Particular attention was directed toward obtaining a control sample representative of the general population from the catchment areas by using a combination of quota and random sampling. Quota sampling segments the catchment area population (using population statistics) to determine the proportion of the local population in certain categories (e.g. gender, age, ethnicity). This is then used to set quotas for the number of controls to be recruited in each category. Two random sampling strategies were then used to identify participants to fill these quotas. First, we used the UK Postal Address File (PAF) – a list of all UK households – as a sampling frame for the catchment area (Jenkins & Meltzer, 1995). A randomly sampled list of addresses in the boroughs of Lambeth and Southwark was generated from the PAF, and each was contacted, in person, three times (morning, afternoon, evening). All eligible adults in each household were invited to take part, and where more than one occupant was willing to participate a modified Kish grid was used to randomly select one member of the household. Second, we used General Practitioner (GP) lists in the catchment area as a sampling frame. Over 95% of individuals in the UK, including inner London, are registered with a GP. We randomly selected 12 GP surgeries within the catchment area and then (via GPs) sent letters to a random sample of 400 individuals in each surgery, inviting them to participate.

Those who agreed to participate completed the Psychosis Screening Questionnaire (PSQ; Bebbington & Nayani, 1995) and were excluded if they screened positive for a psychotic disorder or if they reported a previous diagnosis of psychotic illness.

### Data collection

All subjects were interviewed with a detailed sociodemographic schedule (MRC Socio-demographic Schedule), which was amended to include items on long-term exposure to social adversities, providing information on individual and parental place of birth, individual and parental social class, migration history, ethnicity, housing and living circumstances, current and past addresses, employment history, relationships and social networks (Mallett *et al.* 2002; Morgan *et al.* 2007*b*); the Childhood Experiences of Care and Abuse (CECA) interview to obtain information on separation from or death of a parent/s (Bifulco *et al.* 1994); the Nottingham Onset Schedule to assess duration of untreated psychosis (Singh *et al.* 2005); the Family Interview for Genetics Studies (NIMH, 1992) to collect information on family history of psychosis (indirect measure of genetic risk). We collected clinical data using the OPCRIT (McGuffin *et al.* 1991), information on cannabis and use of illicit drugs using the Cannabis Experiences Questionnaire (Di Forti *et al.* 2009); we calculated IQ using the Satz-Mogel short form of the Wechsler Adult Intelligence Scale-revised (Satz & Mogel, 1962) and premorbid adjustment using the Premorbid Adjustment Scale (Cannon-Spoor *et al.* 1982).

The variables used as indicators of social disadvantage were as follows:

*Childhood disadvantage:* Separation from, or death of, a parent(s) before age 17. For the analyses, we defined long-term separation as a separation (not living in same household) from one or both parents for ⩾6 months resulting from family breakdown (parental separation or divorce, parent abandoned subject). We also focused on changes in family arrangements. Separations consequent upon planned migrations were not included (Table 1).

**Table 1.** Indicators of social disadvantage

**Indicators of social disadvantage in childhood**
1. Separation from, or death of, a parent(s) before age 17
Yes
No
2. Changes in family arrangements before age 17
<2
⩾2
**Indicators of social disadvantage in adulthood**
1. Living status
*Live alone*: alone, alone with children
*Live with relatives*: parents, other family
*Live with others*: partner/spouse, partner/spouse and children, friends, other
2. Relationship status
*Single*: single, divorced, widowed
*In a Relationship*: married, in steady relationship
3. Employment
Unemployed
*Economically inactive*: student, house person, retired, physical illness/disability, career
*Employed*: yes – full time, yes – part-time
4. Housing status
Private owned
Rented
5. Overcrowding
No
Yes
6. Monthly income
Income as median or above
Income below median
Income below official poverty

*Adulthood disadvantage:* We collected data on six social domains (living status, relationship status, employment status, housing situation, overcrowding, monthly income) at the time of the assessment, at onset, 1 year pre-onset and 5 years pre-onset (Table 1). We distinguished between those who lived alone, those who lived with relatives, and those who lived with others, and also, between those who were single and those who were in a relationship. We distinguished between those who were employed, economically inactive and unemployed, and again, between those who were in privately owned accommodation and those who were in a rented accommodation. We defined overcrowding as presence of more persons or couples than bedrooms (separate room for: each couple, single adult aged ⩾21 years, two young people of the opposite sex aged ⩾10 years). We finally distinguished between those who were receiving a monthly income above median (£2119 per month), at or below the UK median and those who received an income below the official poverty line (£1271 per month). We controlled, where possible, for socio-demographic characteristics. To assess the impact of cumulative adult disadvantage, we created indices of current and long-term social disadvantage using, where possible, one indicator variable from each of the domains noted above (i.e. living status, relationship status, employment status, housing situation, overcrowding, monthly income).

### Analyses

To test our main hypotheses, we: (1) Modelled the relationship between each form of childhood and adulthood disadvantage and risk of psychosis, taking account of potential confounding factors, using logistic regression, with case-control status as the main outcome variable. (2) Generated an index of childhood and adulthood adversity in order to assess, using logistic regression, whether the odds of psychosis (case status) increased linearly with extent of childhood and adulthood adversity. In all multivariable analyses, the following variables were controlled: age, gender, and ethnicity. In addition, we further adjusted for each of the following variables (separately, rather than simultaneously due to missing data and consequent reduction of statistical power): place of birth, subject's social class, father's social class, psychiatric family history, family history of psychosis, education, IQ, premorbid adjustment, and use of cannabis. As in previous studies, duration of untreated psychosis was defined as the period in weeks from the onset of psychosis to first contact with statutory mental health services (Singh *et al.* 2005; Morgan *et al.* 2006). Analyses were conducted using Stata v. 11 (Stata, 2009).

## Results

### Sample characteristics

During the study period, 561 cases were identified and approached to participate. Of these, 332 (59.2%) provided informed consent and were included in the study. The reasons why the remaining 229 (40.8%) did not participate included: refused consent, unable to contact again due to discharge from or non-attendance at services, and movement out of the area. We did not have information on those who did not participate and consequently cannot directly assess potential selection bias. However, we were able to compare the basic demographic and clinical characteristics of the 332 included in this study with comprehensive studies of incidence cases (Supplementary Table S1). Broadly, the characteristics of our sample are similar to other incidence samples; CAPsy study has slightly more men, the slight differences in proportions from ethnic groups may reflect demographic shifts in local population over time and/or missing data.

A total of 332 patients with a first presentation of psychosis and 301 controls agreed to participate. Of these, 63% of cases were men and 37% were women, 51% of controls were men and 49% were women. Of the cases, 77% (*n* = 215) had a diagnosis of non-affective psychosis (schizophrenia, schizophreniform disorder, schizoaffective disorder, delusional disorder, psychosis NOS), and 23% (*n* = 64) had a diagnosis of affective disorder (major depressive disorder with psychosis, bipolar I). Differences between cases and controls in age, gender, ethnicity, education and family history of psychosis are described in Table 2. Differences between cases and controls in gender, ethnicity and family history of psychosis reflect well-established associations.

**Table 2.** Basic socio-demographic characteristics by case-control status

	Controls (*n* = 301)	Cases (*n* = 332)	χ^2^/*t*	df	*p*
Age (years)^a^					
Mean (s.d.)	35 (12.32)	28 (8.67)	*t* = 7.43	46	<0.001
Gender^a^, *n* (%)					
Male	153 (50.83)	193 (62.87)	χ^2^ = 8.97	1	0.003
Female	148 (49.17)	114 (37.13)			
Ethnicity^a^, *n* (%)					
White British	131 (43.52)	81 (26.38)	χ^2^ = 25.51	5	<0.001
Asian	15 (4.98)	13 (4.23)			
Black Caribbean	44 (14.62)	53 (17.26)			
Black African	49 (16.28)	81 (26.38)			
Others	24 (7.97)	42 (13.68)			
Non British white	38 (12.62)	37 (12.05)			
Education^b^, *n* (%)					
No qualification	5 (1.67)	53 (17.55)	χ^2^ = 122.06	5	<0.001
School with qualification	133 (11.04)	56 (18.54)			
Tertiary, further	65 (21.74)	52 (17.22)			
Vocational	31 (10.37)	80 (26.49)			
Higher (undergraduate)	105 (35.12)	50 (16.56)			
Higher (postgraduate)	60 (20.07)	11 (3.64)			
Place of birth^a^, *n* (%)					
UK born	196 (65.12)	174 (56.68)	χ^2^ = 4.54	1	0.03
Non-UK born	105 (34.88)	133 (43.32)			
Psychiatric family history^c^, *n* (%)					
No	164 (61.65)	110 (52.13)	χ^2^ = 4.36	1	0.03
Yes	102 (38.35)	101 (47.87)			
Psychosis family history^d^, *n* (%)					
No	252 (95.45)	168 (84.00)	χ^2^ = 17.39	1	<0.001
Yes	12 (4.55)	32 (16.00)			

s.d., Standard deviation; df, degrees of freedom.

Missing values: ^a^25; ^b^32; ^c^156; ^d^169.

### Social disadvantage in childhood

Six per cent (*n* = 20) of patients had experienced loss of a parent due to death before the age of 17, and 6% (*n* = 18) had experienced separation longer than 6 months. Compared to controls, cases were approximately two times more likely to have had a parent die before the age of 17 (OR 1.95, 95% CI 0.98–3.88), and approximately three times more likely to have experienced a long-term separation from one or both parents before the age of 17 (OR 3.04, 95% CI 2.12–4.35) (Table 3). These findings held, with some attenuation, when the ORs were adjusted for age, gender, and ethnicity (Table 3). When we further adjusted (in turn and separately) for other potential confounders such as place of birth, subject's social class (main occupation), father's social class (occupation at birth and lifetime main occupation), education, psychiatric family history, psychosis family history, cannabis use, IQ, premorbid adjustment, the findings held with minimal attenuation (Supplementary Table S2). We also looked at family arrangements, mother and father figures that the subject lived with, for at least 1 year before the age of 17 (e.g. natural mother/father, stepmother/stepfather, etc.), and found that cases were 2.32 times (95% CI 1.66–3.25) more likely than controls to have had ⩾2 family arrangements before age 17 (Table 3).

**Table 3.** Indicators of social disadvantage by case control status

	Controls (*n* = 301), *n* (%)	Cases (*n* = 332), *n* (%)	OR	95% CI	aOR	95% CI
**Childhood**						
Separation, death^a^						
None	176 (59.26)	100 (33.56)	1.00	–	1.00	–
Parental death	18 (6.06)	20 (6.71)	1.95	0.98–3.88	1.80	0.87–3.73
Separation	103 (34.68)	178 (59.73)	3.04	2.12–4.35	2.43	1.64–3.58
Family arrangement^b^						
1	172 (57.91)	110 (37.16)	1	–	1	–
⩾2	125 (42.09)	186 (62.84)	2.32	1.66–3.25	1.75	1.21–2.55
**5 years pre-onset**						
Living status^c^						
Live with others	152 (56.30)	51 (24.88)	1	–	1	–
Live with relatives	50 (18.52)	86 (41.95)	5.12	3.07–8.54	3.24	1.93–5.43
Live alone	68 (25.19)	68 (33.17)	2.98	1.84–4.81	2.66	1.64–4.31
Relationship status^c^						
In a stable relationship	167 (61.85)	94 (45.85)	1	–	1	–
Single	103 (38.15)	111 (54.15)	1.91	1.31–2.78	1.56	1.05–2.32
Employment^d^						
Employed	184 (68.15)	113 (54.85)	1	–	1	–
Economically inactive	67 (24.81)	49 (23.79)	1.19	0.76–1.84	0.86	0.53–1.41
Unemployed	19 (7.04)	44 (21.36)	3.77	2.05–6.91	3.31	1.77–6.20
Housing status^c^						
Privately owned	98 (36.30)	49 (23.90)	1	–	1	–
Rented	172 (63.70)	156 (76.10)	1.81	1.20–2.73	1.32	0.85–2.05
Overcrowding^e^						
No	219 (83.59)	133 (70.74)	1	–	1	–
Yes	43 (16.41)	55 (29.26)	2.10	1.33–3.33	1.51	0.92–2.47
Monthly income^f^						
As median or above	42 (16.80)	8 (4.97)	1	–	1	–
Below median	78 (31.20)	25 (15.53)	1.68	0.69–4.08	1.17	0.46–2.92
Below official poverty	130 (52.00)	128 (79.50)	5.16	2.27–11.75	3.16	1.35–7.43
**1 year pre-onset**						
Living status^g^						
Live with others	168 (55.81)	78 (28.89)	1	–	1	–
Live with relatives	53 (17.61)	92 (34.07)	3.73	2.37–5.89	2.32	1.45–3.72
Live alone	80 (26.58)	100 (37.04)	2.69	1.78–4.05	2.69	1.75–4.13
Relationship status^g^						
In a stable relationship	193 (64.12)	102 (37.78)	1	–	1	–
Single	108 (35.88)	168 (62.22)	2.94	2.06–4.19	2.62	1.82–3.78
Employment^h^						
Employed	208 (69.10)	127 (46.86)	1	–	1	–
Economically inactive	64 (21.26)	59 (21.77)	1.50	0.99–2.29	1.36	0.86–2.14
Unemployed	29 (9.63)	85 (31.37)	4.80	2.90–7.93	4.43	2.67–7.34
Housing status^i^						
Privately owned	116 (38.54)	37 (13.86)	1	–	1	–
Rented	185 (61.46)	230 (86.14)	3.89	2.52–6.02	3.11	1.99–4.86
Overcrowding^j^						
No	253 (85.76)	183 (71.76)	1	–	1	–
Yes	42 (14.24)	72 (28.24)	2.37	1.53–3.65	1.91	1.21–3.02
Monthly income^k^						
As median or above	45 (15.68)	10 (4.44)	1	–	1	–
Below median	100 (34.84)	41 (18.22)	1.84	0.84–4.03	1.48	0.65–3.34
Below official poverty	142 (49.48)	174 (77.33)	5.51	2.61–11.64	3.77	1.72–8.26
**At onset**						
Living status^l^						
Live with others	164 (54.49)	64 (21.99)	1	–	1	–
Live with relatives	55 (18.27)	90 (30.93)	4.19	1.75–5.12	2.43	1.49–3.97
Live alone	82 (27.24)	137 (47.08)	4.28	2.03–5.48	4.62	2.99–7.14
Relationship status^m^						
In a stable relationship	191 (63.46)	83 (30.51)	1	–	1	–
Single	110 (36.54)	189 (69.49)	3.95	2.73–5.72	3.74	2.57–5.44
Employment^n^						
Employed	194 (64.45)	103 (37.73)	1	–	1	–
Economically inactive	64 (21.26)	59 (21.61)	1.73	1.12–2.67	1.71	1.06–2.74
Unemployed	43 (14.29)	111 (40.66)	4.86	3.08–7.66	4.59	2.91–7.25
Housing status^o^						
Privately owned	116 (38.54)	43 (15.99)	1	–	1	–
Rented	185 (61.46)	226 (84.01)	3.29	2.17–4.98	2.61	1.70–4.02
Overcrowding^p^						
No	259 (87.50)	183 (73.20)	1	–	1	–
Yes	37 (12.50)	67 (26.80)	2.56	1.63–4.02	1.96	1.22–3.15
Monthly income^q^						
As median or above	50 (17.30)	10 (4.35)	1	–	1	–
Below median	92 (31.83)	36 (15.65)	1.95	0.88–4.30	1.77	0.78–4.00
Below official poverty	147 (50.87)	184 (80.00)	6.25	2.97–13.16	4.75	2.20–10.28
**At first presentation to psychiatric services**						
Living status^r^						
Live with others	164 (54.49)	56 (18.73)	1	–	1	–
Live with relatives	55 (18.27)	95 (31.77)	5.05	3.10–8.22	2.96	1.80–4.84
Live alone	82 (27.24)	148 (49.50)	5.28	3.39–8.21	5.87	3.77–9.16
Relationship status^r^						
In a stable relationship	191 (63.46)	73 (24.41)	1	–	1	–
Single	110 (36.54)	226 (75.59)	5.37	3.66–7.89	5.31	3.63–7.77
Employment^s^						
Employed	194 (64.45)	64 (21.19)	1	–	1	–
Economically inactive	64 (21.26)	67 (22.19)	3.17	2.00–5.03	3.26	2.01–5.28
Unemployed	43 (14.29)	171 (56.62)	12.05	7.13–20.35	11.15	6.99–17.80
Housing status^l^						
Privately owned	116 (38.54)	43 (14.78)	1	–	1	–
Rented	185 (61.46)	248 (85.22)	3.61	2.39–5.47	2.86	1.86–4.38
Overcrowding^t^						
No	259 (87.50)	203 (77.19)	1	–	1	–
Yes	37 (12.50)	60 (22.81)	2.06	1.31–3.25	1.56	0.97–2.53
Monthly income^u^						
As median or above	50 (17.30)	8 (3.20)	1	–	1	–
Below median	92 (31.83)	25 (10.00)	1.69	0.70–4.06	1.75	0.71–4.30
Below official poverty	147 (50.87)	217 (86.80)	9.22	4.07–20.90	8.35	3.64–19.11

OR, Odds ratio; aOR, adjusted odds ratio for: age, gender, ethnicity; CI, confidence interval.

Missing values (full sample): ^a^38; ^b^40; ^c^158; ^d^157; ^e^183; ^f^222; ^g^62; ^h^61; ^i^65; ^j^83; ^k^121; ^l^41; ^m^60; ^n^59; ^o^63; ^p^87; ^q^114; ^r^33; ^s^30; ^t^74; ^u^94.

### Social disadvantage in adulthood

Across all the domains considered, cases were more likely to report social disadvantage than controls. In particular, at first presentation to psychiatric services, compared with controls, cases were five times (95% CI 3.39–8.21) more likely to live alone, five times (95% CI 3.66–7.89) more likely to be single, 12 times (95% CI 7.13–20.35) more likely to be unemployed, three times (95% CI 2.39–5.47) more likely to live in rented accommodation, two times (95% CI 1.31–3.25) more likely to live in overcrowded conditions, and nine times (95% CI 4.07–20.90) more likely to receive an income below the official poverty line. These associations held after account was taken of age, gender, and ethnicity. These associations were also evident in all domains at onset, 1 year pre-onset and 5 years pre-onset (albeit to lesser degrees) (Table 3).

### Cumulative impact of adult social disadvantage

From the indicators used, we constructed indices of current and long-term social disadvantage in adult life using the following variables: unemployment, living alone, being single, living in rented house, living in overcrowding condition, receiving an income below official poverty.

We dichotomized these variables to indicate the presence or absence of an indicator, with a score of 1 for present (e.g. unemployed) and 0 for absent. This produced a potential range on the current and long-term indices (i.e. at the time of the assessment, at onset, 1 year pre-onset and 5 years pre-onset) of 0–6. It allowed us to investigate whether the odds of psychosis increase in line with increasing disadvantage and whether cumulative effect is present. That is indeed what we found (Table 4). Ninety per cent (*n* = 284) of patients, compared with 60% (*n* = 184) of controls, reported two or more indicators of adult social disadvantage. In other words, cases were around nine times more likely than controls to report ⩾2 indicators of disadvantage at first presentation with psychosis (OR 9.50, 95% CI 5.40–16.70) (Table 4). This association remained when we adjusted for age, gender, and ethnicity (OR 8.05, 95% CI 4.61–14.06).

**Table 4.** Linear relationship and cumulative effect of social adversity by case-control status

	Controls (*n* = 301) *n* (%)	Cases (*n* = 332) *n* (%)	OR	95% CI	aOR	95% CI
Social adversity (5 years pre-onset)^a^						
0	39 (14.44)	9 (4.31)	1	–	1	–
1	66 (24.44)	28 (13.40)	1.83	0.77–4.33	1.11	0.45–2.74
⩾2	165 (61.11)	172 (82.30)	2.95	1.89–4.61	2.14	1.35–3.39
Social adversity (1 year pre-onset)^b^						
0	49 (16.28)	5 (1.82)	1	–	1	–
1	73 (24.25)	31 (11.31)	4.16	1.46–11.81	3.03	1.07–8.58
⩾2	179 (59.47)	238 (86.86)	4.50	2.89–7.00	3.35	2.13–5.27
Social adversity (at onset)^c^						
0	47 (15.61)	3 (1.09)	1	–	1	–
1	70 (23.26)	16 (5.82)	3.58	0.96–13.30	2.58	0.69–9.58
⩾2	184 (61.13)	256 (93.09)	8.56	4.88–15.03	7.09	4.04–12.43
Social adversity (at assessment)^d^						
0	47 (15.61)	4 (1.32)	1	–	1	–
1	70 (23.26)	15 (4.95)	2.51	0.77–8.18	1.82	0.55–5.99
⩾2	184 (61.13)	284 (93.73)	9.50	5.40–16.70	8.05	4.61–14.06

OR, Odds ratio; aOR, adjusted odds ratio for: age, gender, ethnicity; CI, confidence interval.

Missing values (full sample): ^a^154; ^b^58; ^c^57; ^d^29.

**Fig. 1.**
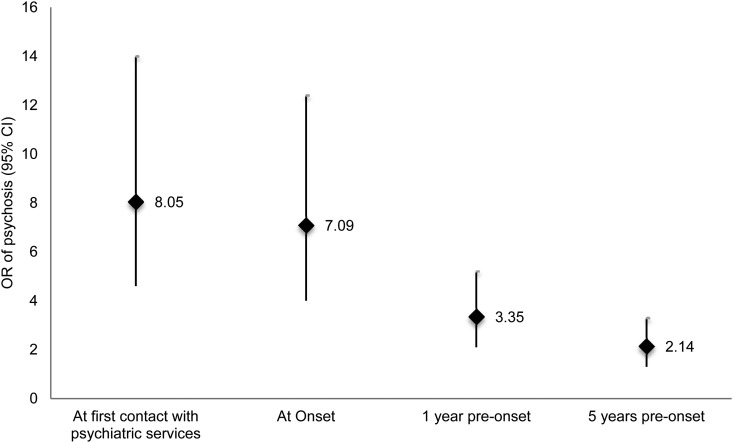
Current and long term effect of cumulative social disadvantage by case-control status. OR, adjusted odds ratio (adjusted for age, gender, and ethnicity).

We repeated these analyses for social disadvantage at onset, 1 year pre-onset and 5 years pre-onset; at all points social disadvantage was strongly associated with later case status. Calculating the adjusted OR, cases were 7.09 (95% CI 4.04–12.43) times more likely than controls to report social disadvantage at onset, 3.35 (95% CI 2.13–5.27) times more likely than controls to report social disadvantage 1 year pre-onset, and 2.14 (95% CI 1.35–3.39) times more likely than controls to report social disadvantage 5 years pre-onset (Fig. 1). The ORs were greater closest to onset suggesting that social disadvantage may not only contribute to onset but may also worsen as a consequence of developing prodromal symptoms. These results were independent of a number of potential confounders (adjusted in turn and separately): place of birth, subject's social class, father's social class, level of education, cannabis use, psychiatric family history, psychosis family history, IQ, and premorbid adjustment (Supplementary Table S3).

### Limitations

Our results should be interpreted in the context of a number of limitations. First, the main issue with case-control designs is bias; cases may have been influenced by their disease experience to recall the nature or the timing of exposures differently from controls by scrutinising their memory for past exposure more intensively than controls (recall bias) and/or the investigators, believing that the exposure causes the disease, may have looked harder for the exposure in cases than controls (observer bias). However, living alone, being single and unemployed are contextual facts that cannot be easily forgotten and our analyses were part of a bigger study with different aims, and many researchers with primary interests unrelated to our hypotheses contributed to the collection of data. Second, although we were able to statistically adjust our results for a series of variables (place of birth, subject's social class, father's social class, level of education, childhood disadvantage, cannabis use, psychiatric family history, psychosis family history, IQ, premorbid social adjustment and premorbid academic performance), the association between social disadvantage and psychosis may be confounded by other unmeasured variables. The fact that the OR for social disadvantage in adulthood increased from 9.5 to 21 when we adjusted for IQ, from 9.5 to 26 when we adjusted for premorbid social adjustment, and from 9.5 to 15 when we adjusted for premorbid academic adjustment, is likely to be the effect of missing data; however, we cannot exclude that these variables may cover the effect of social disadvantage or may interact with social disadvantage. Third, another possible limitation is the definition of ‘social disadvantage’. We considered indicators of social disadvantage in childhood as the presence of separation or loss of one or both parents before age 17 and disadvantage in adulthood as the presence living alone, being single, being unemployed, receiving a monthly income below official poverty, living in rented accommodation, and in an overcrowding condition. As shown by Wicks and colleagues in a Swedish child cohort, housing situation in childhood may be also associated with the risk of developing schizophrenia and other psychoses (Wicks *et al.* 2005). In addition, some of the markers we took as indicating social disadvantage (e.g. living alone) do not always indicate disadvantage while other circumstances may be considered as ‘disadvantaged’; therefore the indicators included must be considered ‘imperfect indicators’. Trauma and life events are without any doubt adverse social experiences and may be linked with the indicators we chose and mutually reinforcing. In addition, for a given individual, the death/separation of a parent, loss of a job, living alone, being single etc. may be considered adverse or not depending on the specific context. Finally, lack of follow-up data did not allow us to establish whether clinical outcome was associated with pre-onset cumulative social disadvantage.

## Discussion

Our findings provide support for the hypothesis that social disadvantages – and the experiences they index – constitute risk factors for psychosis. Consistent with published studies (Agid *et al.* 1999; Morgan *et al.* 2007*b*; Rubino *et al.* 2009; Stilo *et al.* 2013), long-term separation from and death of a parent before the age of 17 were both associated with approximately a 2- to 3-fold-increased odds of psychosis independent of a number of potential confounders. When number of family arrangements was analysed, cases were more likely to report ⩾2 arrangements, a likely effect of separation. All six indicators of social disadvantage in adulthood (living alone, being single, unemployed, living in a rented accommodation, in overcrowding conditions, receiving an income below official poverty) were more prevalent in cases than controls not only at first contact with psychiatric services but also at onset, 1-year pre-onset, and 5 years pre-onset of frank psychosis. The strength of our work is the consideration of long-term associations and cumulative effects of social disadvantage, whereby a greater number of indicators of social disadvantage are associated with greater risk of psychosis. We replicated the findings from the AESOP study (Morgan *et al.* 2008) and the GAP study (Stilo *et al.* 2013) and extended them by using a wider number of indicators of social disadvantage and by looking at social disadvantage at 5 years pre-onset, at 1 year pre-onset, at onset and at first presentation to psychiatric services. In this, our results on childhood and adulthood disadvantage argue against social disadvantage being simply an epiphenomena of impending illness, which is in keeping with other studies (Hafner *et al.* 1999).

As in our previous results (Stilo *et al.* 2013) the strongest factor associated with psychosis was unemployment (OR 12.05, 95% CI 7.13–20.35). Interestingly, by comparing our results with previous studies in England (AESOP collecting data from 1997 to 2000, GAP collecting data from 2005 to 2010) it is evident that the patients currently studied had been experiencing higher levels of social disadvantage compared with controls, a possible consequence of the economic recession which started towards the end of the GAP study (~2008) and intensified during the current study. These changes over time are alarming and social policy should focus on preventing damages caused by current and past social disadvantage.

### Reverse causation

Social disadvantage may be associated with psychosis either because social disadvantage causes psychosis, or because the precursors of psychosis causes social disadvantage. The problem is that causation is not directly observable; it can only be inferred (Schwartz & Susser, 2006). We clearly cannot exclude the possibility that social disadvantage is the consequence of the illness, the consequence of trauma and life events, or that genetic factors may predispose to both social disadvantage and to the illness. However, our data on the duration of untreated psychosis (DUP) suggest that 79% (*n* = 222) of patients had a DUP shorter than 1 year which argues against social disadvantage being simply an epiphenomena of impending illness. Nevertheless, social disadvantage, as described in this article, meets many of Bradford Hill's criteria for causality (Bradford Hill, 1965). In particular we were able to show a strong association between social disadvantage and psychosis (large effect size), consistency (we were able to replicate GAP and AESOP results) (Morgan *et al.* 2008; Stilo *et al.* 2013), temporality (our participants were reporting social disadvantage up to 5 years prior onset and in childhood), risk gradient (greater number of indicators were associated with greater risk), analogy (the social disadvantage–psychosis relationship was analogous to the relationship between other risk factors and psychosis). In addition, although not specifically tested in this article, there are plausible psychological and biological mechanisms that might explain the relationship. Cognitive models for psychosis suggest that pre-existing beliefs and on-going appraisals of experiences are crucial for the development and persistence of positive symptoms of psychosis (Garety *et al.* 2001, 2007; Freeman *et al.* 2002). In this context, social adversity, through negative emotional processes, might contribute to the occurrence and persistence of psychotic symptoms (Freeman *et al.* 2002; Garety *et al.* 2007; Freeman & Garety, 2014).

In addition, evidence of dysregulation of the HPA axis in psychosis has been found in several studies. Neuroendocrinological studies of first episode, drug-naive patients with schizophrenia show evidence of basal over-activity of the pituitary-adrenal axis (Ryan *et al.* 2004; Walsh *et al.* 2005).

As suggested by van Winkel and colleagues ‘behavioural sensitisation’ may play an important role in how psychosocial stress, and social adversities increase the risk for psychosis (van Winkel *et al.* 2008).

The only criteria for causality we could not meet was ‘specificity’ as social disadvantage is not a specific risk factor for psychosis. However, this criterion may not be essential. In relation to physical health, for example, some risk factors are accepted as having a causal role in a number of illnesses (e.g. smoking and lung cancer, heart disease, etc.); that is, they are causal but non-specific.

### Future directions

We found that our sample of cases with psychosis differed from our controls with respect to living status, marital status, work status, housing status, welfare, as well as adversity in childhood such as parental separation, and parental death. We were also able to demonstrate a cumulative effect of disadvantage and a long-term association. However, social disadvantage is only a small part of a much more complex scene.

The question remains of the mechanisms by which social disadvantage may increase risk of psychosis and how social disadvantage combines with genetic liability and other environmental risk factors. Further research will need to focus on the biologically plausible mechanisms by which genes and environment can co-influence onset of disease. Especially, we need to understand how and why genotypes and/or environment confer risk under some conditions but not under others. An interesting approach would be to examine thus, the interplay between the polygenic risk score for schizophrenia and environmental factors.

## Supplementary material

The supplementary material for this article can be found at https://doi.org/10.1017/S0033291716002993.
